# Economic Impact of Dengue Illness and the Cost-Effectiveness of Future Vaccination Programs in Singapore

**DOI:** 10.1371/journal.pntd.0001426

**Published:** 2011-12-20

**Authors:** Luis R. Carrasco, Linda K. Lee, Vernon J. Lee, Eng Eong Ooi, Donald S. Shepard, Tun L. Thein, Victor Gan, Alex R. Cook, David Lye, Lee Ching Ng, Yee Sin Leo

**Affiliations:** 1 Department of Statistics and Applied Probability, National University of Singapore, Singapore, Singapore; 2 Communicable Disease Centre, Tan Tock Seng Hospital, Singapore, Singapore; 3 Saw Swee Hock School of Public Health, National University of Singapore, Singapore, Singapore; 4 Biodefence Centre, Ministry of Defence, Singapore, Singapore; 5 Centre for Health Services Research, National University of Singapore, Singapore, Singapore; 6 Program in Emerging Infectious Diseases, Duke-NUS Graduate Medical School, Singapore, Singapore; 7 Brandeis University, Waltham, Massachusetts, United States of America; 8 Program in Health Services and Systems Research, Duke-NUS Graduate Medical School, Singapore, Singapore; 9 Department of Medicine, School of Medicine, National University of Singapore, Singapore, Singapore; 10 Environmental Health Institute, National Environment Agency, Singapore, Singapore; Pediatric Dengue Vaccine Initiative, United States of America

## Abstract

**Background:**

Dengue illness causes 50–100 million infections worldwide and threatens 2.5 billion people in the tropical and subtropical regions. Little is known about the disease burden and economic impact of dengue in higher resourced countries or the cost-effectiveness of potential dengue vaccines in such settings.

**Methods and Findings:**

We estimate the direct and indirect costs of dengue from hospitalized and ambulatory cases in Singapore. We consider *inter alia* the impacts of dengue on the economy using the human-capital and the friction cost methods. Disease burden was estimated using disability-adjusted life years (DALYs) and the cost-effectiveness of a potential vaccine program was evaluated. The average economic impact of dengue illness in Singapore from 2000 to 2009 in constant 2010 US$ ranged between $0.85 billion and $1.15 billion, of which control costs constitute 42%–59%. Using empirically derived disability weights, we estimated an annual average disease burden of 9–14 DALYs per 100 000 habitants, making it comparable to diseases such as hepatitis B or syphilis. The proportion of symptomatic dengue cases detected by the national surveillance system was estimated to be low, and to decrease with age. Under population projections by the United Nations, the price per dose threshold for which vaccines stop being more cost-effective than the current vector control program ranged from $50 for mass vaccination requiring 3 doses and only conferring 10 years of immunity to $300 for vaccination requiring 2 doses and conferring lifetime immunity. The thresholds for these vaccine programs to not be cost-effective for Singapore were $100 and $500 per dose respectively.

**Conclusions:**

Dengue illness presents a serious economic and disease burden in Singapore. Dengue vaccines are expected to be cost-effective if reasonably low prices are adopted and will help to reduce the economic and disease burden of dengue in Singapore substantially.

## Author Summary

Dengue illness is a tropical disease transmitted by mosquitoes that threatens more than one third of the worldwide population. Dengue has important economic consequences because of the burden to hospitals, work absenteeism and risk of death of symptomatic cases. Governments attempt to reduce the disease burden using costly mosquito control strategies such as habitat reduction and spraying insecticide. Despite such efforts, the number of cases remains high. Dengue vaccines are expected to be available in the near future and there is an urgent need to evaluate their cost-effectiveness, i.e. whether their cost will be justified by the reduction in disease burden they bring. For such an evaluation, we estimated the economic impacts of dengue in Singapore and the expected vaccine costs for different prices. In this way we estimated price thresholds for which vaccination is not cost-effective. This research provides useful estimates that will contribute to informed decisions regarding the adoption of dengue vaccination programs.

## Introduction

Dengue and dengue hemorrhagic fever (DF and DHF, respectively) are substantial public health threats throughout the tropical and subtropical regions [1], [2]. The distribution of dengue and its vectors has expanded dramatically over the last 30 years, among other reasons because of insufficient mosquito control, increasing urbanization and air travel [3], [4]. As a result, about 2.5 billion people worldwide are threatened by dengue infection, with an estimated 50–100 million infections and 12,000 deaths, mainly among children, annually [5], [6].

Determining the disease and economic burden of dengue is crucial in the allocation of scarce public health resources among competing health problems, and to allow for evaluations of the cost-effectiveness of interventions. However, few studies have estimated the economic impact and disease burden of dengue at the national level – while some studies have focused on resource-limited Latin American [7], [8], [9], [10], [11], [12] and Asian countries [10], [13], [14], [15], [16], [17], the broad geographic range of the *Aedes* mosquito vectors also encompasses highly resourced countries and countries that will become highly resourced over the decades ahead. Studies of the health economics of dengue in such settings are scarce, even though the impact of dengue there is substantial.

Singapore presents unique characteristics of dengue infection. Vector control programs introduced in the 1970s led to a considerable decline in vector density and DHF cases [18]; but despite the effectiveness of the vector control programs in reducing vector indices, dengue resurged in Singapore in the 1990s, due to a number of factors chief of which is the reduction of the herd protection in the 1970s and 1980s [19]. As a result, in contrast to other countries in Southeast Asia where dengue is primarily a pediatric disease, over 85% of the reported dengue cases in Singapore are young adults, and the incidence of dengue in the elderly is also growing [18]. Cyclical epidemics have occurred since the 1990s, peaking in 2005 when the incidence of reported confirmed DF was 335 per 100,000 population [20].

Several complexities bedevil the estimation of the economic impact of dengue at the national level. One of the main difficulties is the large proportion of cases that are not reported to national surveillance systems [1]. It is therefore necessary to adjust national statistics using independent cohort or serological studies [21], [22]. Another complexity resides in the heterogeneity of costs: to obtain reliable estimates, it is necessary to combine medical costs with indirect costs borne by the individual, society (e.g. school loss, work absenteeism), and vector control costs. In addition, due to the cyclic nature of dengue epidemics [23], [24], there is no single representative year for dengue infection in a particular region. To stabilize the estimates, projections need to be based on multi-year epidemic cycles [11]. In Singapore, the availability of serological and epidemiological studies independent of the national surveillance system provides a unique opportunity to understand the costs of dengue and allocate resources to control effectively.

At the time of writing, there are tetravalent dengue vaccine candidates in various phases of clinical trials [25], [26], [27], and the determination of cost-effectiveness of these vaccines has been identified as an urgent research need [25]. To address these issues, we performed an estimate of the economic impacts and disease burden of dengue illness in Singapore from 2000 to 2009.

## Methods

### Data collection

Annual national age-dependent DF and DHF cases reported from 2000 to 2009 were obtained from the national surveillance system [28], [29]. Reporting of DF and DHF laboratory diagnosed cases to the Ministry of Health is legally mandated in Singapore. The cases notified by registered medical practitioners and accredited laboratories are collated and totals published weekly by the Communicable Diseases Division of the Ministry of Health [28]. Notification data were complemented with two dengue studies: (a) the prospective Early Dengue (EDEN) Infection and Outcomes study [20], [30] that studied 455 individuals with undifferentiated fever at presentation and (b) the Adult Retrospective Dengue Study at Tan Tock Seng Hospital (ARDENT) that compiled characteristics of dengue patients who presented there from 2004 to 2008. That hospital treated *circa* 40% of all reported dengue cases over this time period.

Epidemic and economic parameters were obtained from EDEN and ARDENT, the literature, official sources and consultation with the National Environment Agency that is responsible for vector control (Tables 1 and 2).

**Table 1:** Epidemic and DALY parameters.

Parameter	Value	Source
Age parameter of logistic model relating age with probability of clinical dengue	0.164†	[33]
Proportion of symptomatic cases using non age-structure rates	0.24–0.53	[34], [35]
*EF_a1_* 15 to 24 years old using age-dependent; constant symptomatic rates	3.8; 1.7–3.6	Estimated from [32], [33], [34], [35]
*EF_a2_* 25 to 34 years old using age-dependent; constant symptomatic rates	13.1; 3.8–8.2	Estimated from [32], [33], [34], [35]
*EF_a3_* 35 to 44 years old using age-dependent; constant symptomatic rates	24.3; 6.1–13.4	Estimated from [32], [33], [34], [35]
*EF_a1_* 45 to 54 years old using age-dependent; constant symptomatic rates	45.3; 11.1–24.2	Estimated from [32], [33], [34], [35]
*EF_a1_*>55 years old using age-dependent; constant symptomatic rates	50; 12.2–26.5	Estimated from [32], [33], [34], [35]
Expansion factors for hospitalised cases, *EF_h_*	1.4–3.4	[22], [31]
Number of ambulatory visits per episode	4.33	ARDENT project
Average length of hospitalization (days)	4.6–4.8	[20], [59], ARDENT project
Number of fatalities from 2000 to 2009.	98¥	[29]
Disability weight for symptomatic cases of DF from WHO and the literature, *D*	0.211; 0.81	[9], [43]
Disability weight for symptomatic cases of DHF from WHO and the literature, *D*	0.5; 0.85	[42], [43]
Mean disability weight for symptomatic ambulatory and hospitalized children cases, *D*	0.37; 0.52§	[44]
Mean disability weight for symptomatic ambulatory and hospitalized adult cases, *D*	0.42; 0.53§	[44]
Social discount rate for DALYs calculations, *r*	0.03	[41], [60]
Age-weighting correction constant, *C*	0.16243	[41]
Parameter of the age-weighting function, *β*	0.04¶	[41]
Duration of disability in reported cases (days)	10.4	[20], [59], ARDENT project
Duration of disability in unreported cases (days)	4	[16]
Duration of disability in DHF cases (days)	14	[11], [61]
Proportion of cases reported that are hospitalized	0.565	[20], [59], ARDENT project
Proportion of hospitalized cases that are DHF	0.358	ARDENT project

**†:** The intercept of the linear model was estimated to be −2.94 [33].

**§:** Estimated from Figure 1 in [44] for 10 days of symptoms.

**¥:** Deaths per year: 2000: 2; 2001: 6; 2002: 6 ; 2003: 6 ; 2004: 8 ; 2005: 27 ; 2006: 10; 2007: 24 ; 2008: 2 ; 2009: 8. No expansion factors were applied to the number of deaths.

**¶:** The age weighting function represents the value of life at different ages. It reflects the different social roles of individuals at different ages, i.e. young and elderly require care giving [41].

**Table 2:** Economic parameters (2010 US $).

Parameter	Value	Source
Hospital costs per hospitalized case per day ($)	Normal(431,597)*	[36]
Transport costs to seek medical care and household members visiting patients ($)	3.7†	[62]
Average costs per ambulatory visit ($)	62.1§	ARDENT project
Average productivity loss per absent day of work in individuals from 18 to 64 years ($)	163‡	[63]
Average household services losses per day ($)	35¢	[40]
Elasticity of annual labour time versus labour productivity	Uniform(0.6,0.9)	[38]
Proportion of children that require a parent to be absent from work for care giving	0.43¥	[64]
Proportion of elderly needing to hire a care giver	0.073¶	[65]
Cost of providing primary education per student per day	21∥	[39]
Cost of providing secondary education per student per day	29.3∥	[39]
Discount rate for premature deaths productivity lost	0.03	[60]
Vaccine effectiveness (%)	80††	[53]
Vaccine overhead, labour, syringes, distribution and storage costs per dose ($)	7◊	[52]
Annual expenditure on dengue control ($ million)	50	NEA

*Estimated using the bill sizes per dengue patient and day. The distribution was truncated to only positive values.

**†:** Average daily ridership and average round trip distance used to calculate weighted average transportation cost. It includes Mass Rapid Transport and Light Rapid Transport systems, bus, and taxi. An average of two family visits per day per inpatient are assumed. Transport in Singapore is not subsidized [66].

**§:** Includes the costs and proportion of patients tested using dengue PCR ($111.5) or serology tests ($25.9) in the first consultation , medical officer consultation fees ($30.9 for first consultation and $24.3 subsequent consultations), cost of full blood count in all consultations ($16.4), urea ($6.7), protein ($7.1), ALT ($7.1) and AST ($7.1) tests and cost of symptoms relief medicaments ($6.6 including paracetamol for fever, metocloperamide for vomiting, peritoh for itch and famotidine for gastric irritation).

**‡:** Obtained by dividing the GDP per capita by the working days per year. The productivity loss by an undetermined day of work is obtained by dividing the GDP per capita by 365 days.

**¢:** Due to lack of data on allocation of hours to household activities in Singapore, we employ US cost data expressed in US 2010 $. In the model we distinguish the household service losses per day in the age groups: 15–17 ($15), 18–29 ($26), 30–64 ($40), 65–74 ($45) and >75 ($38).

**¥:** Families in this situation are assumed to be families with all working parents, without maid and without unemployed or retired family members available to give care to the children. The proportion of children belonging to families where nobody cooks at home is used as a surrogate for these families [64] . For the rest of the families, the care giver is imputed a cost corresponding to the household services that cannot be carried out during the time of care giving.

**¶:** The estimate corresponds to the number of persons >65 years old living alone. A social worker with a salary of $13/hour is assumed to be hired as care giver 8 hours a day. For the rest of elderly the care givers are imputed a cost equal to their household services.

**∥:** Average government expenditure divided by total number of primary or secondary students and total school days.

**††:** Low effectiveness as compared to previous studies assuming 95% [53] to reflect the difficulty of obtaining a vaccine for the four serotypes.

**◊:** Corresponds to Panama in [52] .

### Degree of underreporting

Underreporting was corrected using expansion factors [21] (EF) to scale reported cases. As more severe cases, such as those hospitalized, are much more likely to be reported than mild cases treated in ambulatory care, we distinguished between two expansion factors: *EF_h_* for hospitalized cases (*EF_h_* was conservatively estimated from the lower bound estimates from the literature [22], [31]); and *EF_ai_* for ambulatory cases in age group *i*. To estimate *EF_ai_* for different age groups, we employed the results from a serological study in 2004 among 18 to 74 year olds as part of the National Health Survey [32]. The sampling was considered representative of the population because participants were recruited from different sentinel sites across the country, and selected by a combination of stratified and systematic sampling. The study results were used to infer total prevalence of dengue infection in each age group. The total dengue symptomatic prevalence in each age group was then estimated by multiplying the total number of serologically identified dengue cases by symptomatic rates. Given the uncertainty regarding symptomatic rates, we considered two main scenarios: (i) an age-dependent symptomatic rate [33]; and (ii) a constant range of symptomatic rates [34], [35]. Seroconversion for children during that period was not available and we therefore assumed that the expansion factor for the young adults applied also for children.

### Direct costs

We considered both medical and non-medical direct costs. Direct medical costs were calculated for hospitalized and ambulatory cases. Daily hospitalization costs were obtained from the distribution of hospital bills per dengue patient provided by public Singaporean hospitals in 2010 for unsubsidized wards, divided by the median length of stay [36]. The median and 90^th^ percentile daily costs per patient were used to construct a normal distribution (Table 2). The costs of ambulatory cases were obtained by multiplying the average number of visits per case by the unit costs of each visit (Tables 1 and 2). The costs included consultation fees, tests performed, and treatment costs (Table 2).

Non-medical direct costs include individual and family transport costs (Table 2), and control costs which were obtained from the National Environment Agency. All costs were expressed in 2010 US dollars.

### Indirect costs

Indirect costs were expressed per unspecified day and included reduction of work productivity, reduction of household services, loss of schooling, and increased need for caregivers. To estimate work productivity loss, the World Health Organization (WHO) proposes two main methods, both of which we used: the human capital and the friction cost method [37]. The human capital method values lost time or premature death using the individual's gross earnings, derived from the gross domestic product per capita. The more conservative (lower cost) friction cost method acknowledges that job absenteeism or death lead to productivity losses that can be temporarily offset by colleagues or by hiring new labour [38], so that the loss of productivity occurs only during a friction time period (assumed to be in our case 30 days for fatalities and to last as long as symptoms in non-fatal cases) and productivity losses are offset according to the elasticity of annual labour time versus labour productivity (Table 1). Friction costs were then calculated by multiplying the length of the friction period with the expected average gross earnings in the period and the elasticity of annual labour time versus labour productivity. The costs of school days lost were estimated from the expenditures on schools in Singapore per student per day [10], [39].

We also estimated the impact on household services, which are not paid for but represent important economic activity (e.g. cleaning, cooking, caring for children and the elderly) (Table 2) [40]. Losses of household services affect not only the working population but also the young and the elderly [40].

We assumed that symptomatic children with two working parents but without household help caused further job absenteeism. For the elderly, only those outpatients living alone were assumed to require a caregiver (Table 2). For cases where care was given by a member of the family not actively working, the care givers incurred a loss of household services.

### DALYs estimation

Different disability weights for DF and DHF have been used in previous studies. For comparison, we employ three sets of disability weights: the first, based on recent literature estimates, reflects that all symptomatic cases are incapable of carrying out normal daily activities during illness [9], [16], [41], [42]; the second based on WHO disability weights [43]; and the third has weights obtained in a empirical study that measured daily the losses in quality of life through the course of the infection using the visual thermometer-like scale technique [44], [45] (Table 1). A disability weight of 1 was used for premature death. DALYs lost by each case were calculated using [41]:
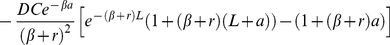
where *D* is the disability weight; *r* is the social discount rate; *a* is the age of the individual at the onset of symptoms; *L* is the duration of the disability or the years of life lost due to premature death expressed in years; *C* is the age-weighting correction constant; and β is the parameter from the age-weighting function. The age-weighting function represents the value of life at different ages [41] (Table 1).

### Vaccine cost-effectiveness

Because the eventual price of the vaccine is very uncertain, instead of assuming one single price we estimated the threshold price above which vaccination programs of different characteristics would not be cost-effective [25]. We compared the cost-effectiveness of the vaccines with the current vector control program ($4,740 per DALY averted [46] ) and the criterion for cost-effective health interventions of WHO (cost per DALY averted below 3 times the gross national income per capita [47]).

We considered a scenario of mass vaccination allocated at random to a proportion of the population. The vaccination program could require two or three doses and could confer lifetime or only ten years immunity, leading to a total of four combinations of vaccine characteristics.

Vaccine cost-effectiveness was evaluated for a time period of 75 years equivalent to the country's average life expectancy. Average annual estimates of DALYs and economic impacts were estimated per capita for each age group from 2000 to 2009 and used to project economic impacts and DALYs using the population levels and age structure in Singapore as predicted in the United Nations World Population Prospects 2010 Revision from 2012 to 2086 [48]. By 2086, Singapore is expected to increase its population from 5.3 million of 6.5 million and to increase the proportion of habitants above 65 years old from 11% to 40% [48].

To estimate the critical vaccination coverage (*f_c_*) we considered the largest dengue epidemic in Singapore during the last 10 years, which occurred in 2005 [49] . It has been estimated that the basic reproductive number (R_0_, where an outbreak with an R_0_ below 1 dies out naturally [50]) fell in the range 1.89–2.23 [49]. The vaccine coverage *f_c_* to bring the basic reproduction number *R_0_* below 1 with a vaccine of efficacy γ is:
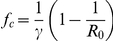
we assumed that vaccine programs attaining herd protection greater than or equal to *f_c_* would prevent epidemics of dengue in Singapore (ignoring localised non-sustainable outbreaks following importation).

## Results

### Underreporting

The serological study in 2004 tested for IgG and IgM antibodies among 4152 individuals. Of the study population, 59.0% and 2.6% tested positive for dengue IgG and IgM that are indicative of past and recent infection (within the last three months), respectively. The rate of recent infection ranged from 1.2% in individuals from 15 to 24 years old to 3.2% in individuals from 45 to 54 years old [32]. We assume that the number recently infected in the time period of the study is representative of the proportion infected in the country for that time period. From the 2004 population age structure, we estimated that 71,134 individuals were recently infected – encompassing symptomatic and asymptomatic cases – nationally in the period of the study. The number of reported cases during the same time period was 3104. To obtain the number of symptomatic infected individuals, we multiplied the estimated number of individuals recently infected with symptomatic rates. Due to uncertainty in the asymptomatic rates in each age group, we considered two scenarios to obtain expansion factors. In the first scenario, we multiplied the expected number of infected individuals with age-dependent symptomatic rates obtained from a logistic model [33]. In a second scenario, we multiplied by a range of constant symptomatic rates for all ages of 24% to 53% [34], [35]. We obtained two sets of expected number of infected symptomatic cases per age group, and compared this with the cases reported per age group. In the first scenario, the expansion factors ranged from 3.8 in the youngest group (0–24 years) to 50 in the oldest group (>55 years) (Table 1). The second scenario yielded expansion factors ranging from 1.7–3.6 for 0–24 years to 12.2–26.5 for >55 years. The proportion of underreporting increased with age in both scenarios.

### Economic and disease burden

**Figure 1:**
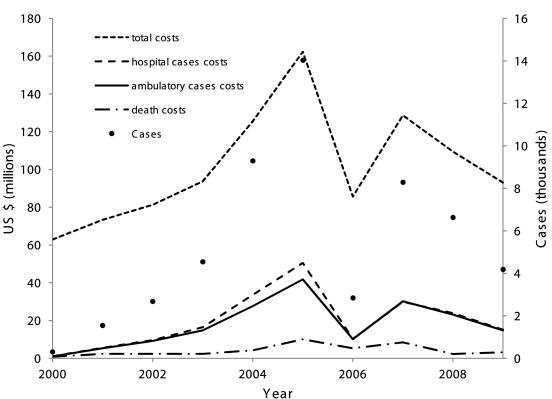
Economic impacts of dengue in Singapore. Mean total economic impacts, costs from hospitalized cases, costs from ambulatory cases and from fatalities due to dengue in Singapore from 2000 to 2009 using the human capital method and constant symptomatic rates.

The mean economic impact was mostly driven by the number of cases per year, resulting in high variability (Figure 1). For instance, combining the human capital method and non-age-dependent symptomatic rate scenarios during the 2005 epidemic led to costs of US $160 million, more than double the cost in 2000 ($64 million, Figure 1).

Using the human capital method and non-age-dependent symptomatic rates, the distribution of costs from 2000 to 2009 excluding control costs had a mean of $415 million ($41.5 million per year) with 5^th^ and 95^th^ percentiles of $299 and 569 million (Table 3). Using the friction cost method, the mean was $351 million with 5^th^ and 95^th^ percentiles of $236 and 504 million. Total control costs were $500 million. Hence the total economic costs from 2000 to 2009 were $0.91 billion using the human capital method or $0.85 billion using the friction cost method. Using age-dependent symptomatic rates, the total cost estimates increased to $1.06 billion by the friction cost method and $1.15 billion by the human capital method (Table 3). The costs due to deaths decreased considerably under the friction cost method (Table 3). Whereas age-dependent symptomatic rates led to a higher proportion of costs due to ambulatory cases, hospitalized cases represented the largest share of costs when constant symptomatic rates were used (Table 3). The relative percentage of costs due to hospitalized cases and deaths decreases with respect to ambulatory costs when considering age-dependent symptomatic rates (Table 3, columns 5^th^ and 6^th^). The reason is that, whereas using age-dependent symptomatic rates leads to higher expansion factors estimated for ambulatory cases than using constant symptomatic rates, the number of fatalities and the expansion factors for hospitalized cases does not vary.

**Table 3:** Economic impacts of dengue in Singapore.

Method	Symptomatic rate	TC ($ billion) without control	% ambulatory costs	% hospitalized costs	% death costs	% costs lost productivity	TC ($ billion) with control
Human capital	Constant	0.41 (0.30; 0.57)	43	47	10	24	0.91
	Age-dependent	0.65 (0.53; 0.80)	62	32	6	29	1.15
Friction cost	Constant	0.35 (0.24; 0.50)	47	53	0.2	21	0.85
	Age-dependent	0.56 (0.45; 0.72)	67	34	0.1	25	1.06

Using empirically derived disability weights [44], average DALYs per 100,000 population were 8.7 (5^th^ and 95^th^ percentiles of 8 and 10) when using constant symptomatic rates and 14 (5^th^ and 95^th^ percentiles of 13 and 16) when using age-dependent symptomatic rates (Table 4). DF made up 24–32% of the disease burden, non-fatal DHF 33–57%, and dengue related deaths 9–43% (Table 4). For comparison with previous studies we repeated the analysis with disability scores from WHO [51] (Table 4, 8–8.9 DALYs per 100,000 population) and with literature disability scores (16–27 DALYs per 100,000 population).

**Table 4:** Disease burden of dengue in Singapore.

Disability weights	Symptomatic rate	Total disease burden (DALYs/100000)	% DF DALYs	% DHF DALYs	% death DALYs
From literature [9], [16]	Constant	16.0 (13.2; 18.0)	31	54	16
	Age-dependent	27.4 (23.3; 31.3)	34	57	9
From WHO [43]	Constant	5.8 (5.2, 6.5)	24	33	43
	Age-dependent	8.9 (7.9; 9.9)	26	49	25
Empirically derived [44]	Constant	8.7 (7.7; 9.9)	27	45	28
	Age-dependent	14.4 (12.6; 16.4)	32	50	18

### Vaccine cost-effectiveness

We conservatively evaluated the cost-effectiveness of vaccines using constant symptomatic rates and empirically derived disability weights. Assuming the worst dengue epidemic of *R_0_* = 2.5 and a vaccine of efficacy γ = 0.8 (to reflect the difficulty to obtain a vaccine effective to the four serotypes), the critical herd protection needed against the four serotypes to prevent dengue epidemics (*f_c_*) would be 75%. The actual herd protection in Singapore is uncertain. Under the conservative assumption of a completely dengue-naïve population, a general vaccination program covering 75% of the population would be expected to prevent dengue epidemics within one year of completion.

**Figure 2:**
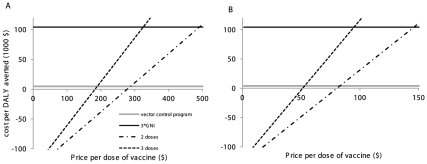
Cost-effectiveness of dengue vaccines. Cost per DALY averted for mass vaccination programs requiring 2 and 3 doses. A: the vaccine confers lifetime immunity; B: the vaccine confers 10 years immunity. 3*GNI indicates the trice of the gross national income per capita. A comparison between the costs per DALY averted of the different vaccination programs with the current cost per DALY averted of the vector control program (“vector control program”) and the cost-effectiveness threshold (3•GNI) is made. When the costs per DALY averted are higher than the costs per DALY averted of the vector control program, the vector control program is comparatively more cost-effective. If the costs per DALY averted are higher than 3•GNI, the vaccination program is not cost-effective for Singapore.

Conservatively assuming that vector control costs remain constant, we evaluated the vaccine programs' cost-effectiveness with increasing vaccine prices (Figure 2). The threshold price beyond which vaccines would not be cost-effective increased when fewer doses were needed and longer immunity was conferred. For low prices, vaccines presented net savings per DALY averted (avoided costs were greater than vaccination costs) and were very cost-effective. The price per dose threshold beyond which vaccines stopped being more cost-effective than the current vector control program ranged from $53 for mass vaccination requiring 3 doses and only conferring 10 years of immunity to $287 for vaccination requiring 2 doses and conferring lifetime immunity (Figure 2 A). The thresholds for vaccine program cost-effectiveness in Singapore ranged from $95 and $491 per dose respectively (Figure 2 B). For sensitivity analysis purposes, assuming instead that the population size and age structure remained constant in the future in Singapore, the thresholds for these vaccine programs to not be cost-effective in Singapore were lower ($70 and $212 respectively) due to their lower potential avoidance of the economic burden of dengue.

### Sensitivity analysis

We evaluated the sensitivity of the mean estimated disease burden, total costs and the benefit-cost ratio of the vaccination programs to the model parameters considering both ten-year and lifelong immunity. We performed univariate sensitivity analysis where all parameters were increased by 30% to evaluate their relative importance. The analysis showed that disease burden estimates were sensitive to the parameters: length of symptoms of DHF cases (increase of 16%), disability weight for DF cases (increase of 19%) and the proportion of DHF cases (increase of 10%). The total cost estimations were sensitive to the expansion factor used for hospitalized cases (increase of 14%), hospitalization costs per day (increase of 11%) and length of hospital stay (increase of 10%).

The benefit-cost ratios of a mass vaccination program conferring lifelong immunity decreased when increasing the discount rate used (18%), costs of overhead, vaccine storage and distribution (22%), the number of doses needed (28%) and the required herd protection to drive R_0_ below 1 (27%). The same direction in sensitivity was obtained for vaccine programs conferring ten-year immunity. However, the magnitude of the effects increased by 5%, on average, compared to the estimates for lifelong immunity.

## Discussion

The burden of disease due to dengue infections is high across at-risk areas of the world. Even with good vector control, as Singapore has, permanent reduction of dengue epidemics has proven to be impossible, and vaccines may be the only hope for sustained control. Our analysis demonstrates that dengue imposes a significant disease and economic burden in Singapore. The cost-effectiveness of vaccines will depend on their price and characteristics.

To be able to estimate how cost-effective the vaccines will be, a baseline price can be used. Considering a price per dose of $5 (based on the projected price of a dose of pneumococcal vaccine) from a cost-effectiveness study for dengue vaccines in Panama, a middle income country [52], all the vaccination programs considered would be very cost-effective and would provide net savings per DALY averted, which is in stark contrast with current costs of $4,740 per DALY averted by the vector control program [46] and with a vaccine cost-effectiveness evaluation of $50 per DALY averted with prices of routine vaccines in resource-limited settings ($0.50 per dose in the public sector [53]). However, the price of recently developed vaccines in Singapore is much higher (e.g. US $124 per dose of pneumoccal vaccine for 3 required doses) [54]. If we use the considerably higher price of US $124 per dose as the baseline price, for mass vaccination to be cost-effective, it would have to guarantee lifetime immunity. At this high price the vaccination programs involving 3 doses and conferring only 10 years of immunity would not be more cost-effective than the vector control program (Figure 2); however, the other programs involving lifetime immunity or only two doses would be more cost-effective than vector control. The comparison with the cost-effectiveness of the vector control program, however, is only illustrative: a vaccination program might still be preferred as long as the cost per DALY averted is below three times the gross national income per capita, since deaths due to dengue will be avoided and they would have been unavoidable under the current vector control program. This reflects the importance of the substantial incremental costs of the vector control program to attain lower than current disease burdens. Using three times per capita gross national income as the cost-effectiveness threshold [47], the price threshold of the vaccines is very high. For instance, a vaccine involving three doses and conferring only ten years of immunity would be cost-effective up to a price threshold of $95 per dose (Figure 2).

Our results on total costs were sensitive to hospitalization costs. This reflects the high hospitalization costs of Singapore relative to other South East Asian countries, e.g. Thailand, where non-hospitalized cases represented a substantial proportion of the overall burden of the disease [13]. Ambulatory cases, however, also represent a large share of the total costs due to dengue in Singapore (Table 3).

The disease burden of dengue in Singapore (9–14 per 100,000 population) using empirically derived disability weights is comparable to diseases like hepatitis B or syphilis (10 and 9 DALYs per 100,000 respectively). Using disability weights from the literature [9], [16] dengue is comparable to meningitis and multiple sclerosis (22 and 19 DALYs per 100,000, respectively, versus our estimated 16–27) [43]. It is, though, lower than other tropical and subtropical countries (e.g. 66 in Puerto Rico [9], 42.7 in Thailand [16] and 26.5 DALYs per 100000 in Brazil [11], where the estimates were obtained using the same disability weights from the literature). Different estimates were also obtained when using WHO disability weights (Table 4), and consensus would be necessary for results to be comparable across studies.

The lower disease burden per capita in Singapore compared to other studies may be due to its intensive vector control program, which represents the greatest component of dengue costs (42–59%). This may indicate that vector control in Singapore is attaining its maximum expected effectiveness. Given the high endemicity levels of dengue in Southeast Asia and the constant movement of persons and commodities between the countries in the region, increasing the efforts in vector control would likely meet with diminishing returns in dengue incidence. Hence, an effective dengue vaccine remains an attractive option for long-term and sustainable dengue prevention. We found that for reasonably low prices, vaccines are a promising and cost-effective option to reduce cases further. However, the extent to which vaccination might reduce necessary vector control expenditures is unknown, as vector control would still be necessary to prevent outbreaks of other mosquito-borne diseases e.g. chikungunya, which reached Singapore in 2008 [55]. On the other hand, if vector control activities were reduced as a result of the vaccination program, the cost-effectiveness of the vaccines would be higher. We preferred, however, to adopt a conservative approach by considering no reductions in the costs of the vector control as a result of the vaccination program. At the same time, improvements in vector control technology such as application of genetic modification techniques to the Sterile Insect Technique [56] or the introduction of the bacterium *Wolbachia* in mosquito populations [57] might be attractive alternatives or complements to vaccination, especially when the timeline for availability of vaccines, their eventual efficacy and length of protection are unknown.

The main limitations of the study reside in the presence of uncertainty regarding key parameters. For instance, the vaccine might be less effective than assumed and be associated with high post-implementation costs. These factors would reduce the price threshold for which the vaccine would be cost-effective, but given the large margin of error for the vaccine to be cost-effective and the conservative approach adopted, we are confident that for reasonably low prices, the vaccine will be cost-effective in Singapore.

We have not evaluated the cost-effectiveness of purely pediatric vaccines since their implementation would involve only partial protection of the population, and to estimate their cost-effectiveness would require an epidemic model capturing the dynamics of dengue in Singapore and able to relate partial population immunity with disease prevalence would be necessary. The construction of such an epidemic model would be a complex undertaking given the high uncertainty regarding the mechanisms that drive dengue dynamics in Singapore, and so this was left for future research. We postulate however that pediatric vaccines are likely to be also cost-effective [53] although it might take 10 to 20 years to notice their effect on disease burden reductions.

The estimation of the economic and disease burdens also presented limitations. We were unable to estimate the intangible costs due to the extra burden of dengue epidemics to the health system; we also could not find a significant relationship between dengue cases and volume of tourism or other economic sectors in Singapore. The exclusion of these economic impacts makes our estimate of the economic burden conservative. Uncertainty was also present in the estimation of underreporting, or expansion factors. We were unable to estimate expansion factors for hospitalized cases and had to rely on existing literature. To gauge the lower bound of our estimates, assuming that all hospitalized cases are reported (EF_h_ = 1), the total costs would be reduced by 18%. For ambulatory cases, the availability of national serological surveys compared with nationally reported cases gives strong confidence in our estimates. The symptomatic rate estimates however, presented high variability per age group and were scarce in the literature, leading to rather different disease burden estimates. To account for this uncertainty, two scenarios were considered, with broadly similar findings. Nonetheless, further research on symptomatic rates per age group would be beneficial to derive future estimates. Using age-dependent symptomatic rates, our estimates of expansion factors for age groups below 44 years old (3.8, 13.1 and 24.3) were approximately equivalent to those in other studies, e.g. Brazil (2.1–10), Colombia (4.5–18) or Puerto Rico (10–27) [11] but were higher in older age groups (45.3 and 50). Using constant asymptomatic rates, the expansion factors matched these estimates from the literature. Comparison between studies is difficult because age-dependent expansion factors for multiple age-groups are rarely calculated. One exception is Meltzer et al. [9], who estimated expansion factors of 10 for 0–15 years old and 27 for cases above 15 years old, which is in agreement with our results regarding increasing underreporting with age. The reason for underreporting increasing with age might be due to parental influence for the young [9] and/or atypical disease manifestations of the elderly [58].

In summary, we demonstrated the high economic and disease burden of dengue in Singapore and our results strongly support the implementation of vaccination programs if reasonably low prices are adopted. Vaccines will assist in Singapore as a mean to curb the economic and health burden of dengue illness.
